# Increased Yield of Extracellular Vesicles after Cytochalasin B Treatment and Vortexing

**DOI:** 10.3390/cimb45030158

**Published:** 2023-03-15

**Authors:** Sirina V. Kurbangaleeva, Valeriia Y. Syromiatnikova, Angelina E. Prokopeva, Aleksey M. Rogov, Artur A. Khannanov, Albert A. Rizvanov, Marina O. Gomzikova

**Affiliations:** Institute of Fundamental Medicine and Biology, Kazan Federal University, Kazan 420008, Russia

**Keywords:** extracellular vesicles, cytochalasin B-induced membrane vesicles, mesenchymal stem cells, delivery system

## Abstract

Extracellular vesicles (EVs) are promising therapeutic instruments and vectors for therapeutics delivery. In order to increase the yield of EVs, a method of inducing EVs release using cytochalasin B is being actively developed. In this work, we compared the yield of naturally occurring extracellular vesicles and cytochalasin B-induced membrane vesicles (CIMVs) from mesenchymal stem cells (MSCs). In order to maintain accuracy in the comparative analysis, the same culture was used for the isolation of EVs and CIMVs: conditioned medium was used for EVs isolation and cells were harvested for CIMVs production. The pellets obtained after centrifugation 2300× *g*, 10,000× *g* and 100,000× *g* were analyzed using scanning electron microscopy analysis (SEM), flow cytometry, the bicinchoninic acid assay, dynamic light scattering (DLS), and nanoparticle tracking analysis (NTA). We found that the use of cytochalasin B treatment and vortexing resulted in the production of a more homogeneous population of membrane vesicles with a median diameter greater than that of EVs. We found that EVs-like particles remained in the FBS, despite overnight ultracentrifugation, which introduced a significant inaccuracy in the calculation of the EVs yield. Therefore, we cultivated cells in a serum-free medium for the subsequent isolation of EVs. We observed that the number of CIMVs significantly exceeded the number of EVs after each step of centrifugation (2300× *g*, 10,000× *g* and 100,000× *g*) by up to 5, 9, and 20 times, respectively.

## 1. Introduction

All mammalian cells release extracellular vesicles (EVs)—spherical particles from 30 nm to 1 µm in diameter surrounded by a double phospholipid layer [1]. The functions of EVs are the mediation of intercellular communication and delivery of the membrane and cytosolic components (proteins, lipids, and nucleic acids) of host cells [2,3]. EVs are involved in many physiological and pathological processes, acting as mediators of intercellular communication [4]. The composition of the “cargo” of extracellular vesicles depends on the type of the parent cell and signals from the extracellular environment that trigger the release of EVs. The contents of EVs include lipid mediators (eicosanoids), proteins (cytokines, chemokines, growth factors, or other signaling mediators), genetic material (mRNA, snRNA, long/short non-coding RNA, and nuclear and mtDNA), and, in the case of larger vesicles, whole organelles (for example, mitochondria) [5,6,7,8,9,10,11].

It has been shown that EVs derived from mesenchymal stem cells demonstrate parental cell activity: they stimulate cell viability [12], wound healing [13], and repair of damaged tissue [14], induce angiogenesis [15], and have an immunomodulatory effect [16]. Therefore, EVs derived from MSCs (EVs-MSCs) are considered a promising therapeutic tool [17]. In addition, EVs-MSCs have the following advantages: (1) they do not express MHC-2 and show little expression of MHC-1 complexes, therefore, they are non-immunogenic [18]; (2) EVs’ contents are protected from degradation by a cytoplasmic membrane; (3) EVs are unable to divide and, therefore, are a safer therapeutic tool than MSCs themselves; and (4) EVs are smaller in size compared with parental cells and show good biodistribution in vivo (for example, EVs are 100 nm in size while MSCs are 16,900 nm in size) [19,20,21,22].

Despite the high therapeutic potential, the wide introduction of EVs into clinical use is limited due to the small yield of vesicles. To date, a large number of methods for the large-scale production of EVs have been developed based on bioreactor culturing, the modulation of culture conditions, chemical stimulation, physical stimulation, or cell membrane disruption [23]. In our study, we used the chemical treatment of cells with cytochalasin B, which was first described by Pick et al [24]. Cytochalasin B blocks the polymerization of actin microfilaments leading to the disorganization of cell cytoskeleton, which is a necessary condition for the detachment of EVs from the cytoplasmic membrane [23]. Then, to induce the pinching off of membrane vesicles, active vortexing is applied [24]. The induced vesicles obtained in this way include functional receptors of the plasma membrane and cytosolic molecules of the donor cells [24]. Previously, we showed that cytochalasin B-induced membrane vesicles (CIMVs) have a similar content and immunophenotype as parental MSCs and demonstrated their angiogenic activity [25,26]. We compared the immunomodulatory effect of MSCs, CIMVs, and EVs, and observed no immunosuppression in mice pretreated with natural EVs, whereas MSCs and CIMVs-MSCs suppressed antibody production in vivo [27].

However, no comprehensive comparative analysis of natural EVs and those induced with cytochalasin B membrane vesicles has been carried out so far. Therefore, in this study, we sought to characterize, determine the yield of, and conduct a comparative analysis of the natural EVs and CIMVs derived from MSCs.

## 2. Materials and Methods

### 2.1. MSCs Isolation

MSCs were isolated from murine adipose tissue. The adipose tissue was incubated in 0.2% collagenase II (Dia-M, Moscow, Russia) solution for one hour in a shaker–incubator at 37 °C, 120 rpm. The cell suspension was pelleted (400× *g* for 5 min), washed thrice in PBS (PanEco, Moscow, Russia), and re-suspended in DMEM/F12 (PanEco, Moscow, Russia) supplemented with 10% fetal bovine serum (Sigma-Aldrich, St. Louis, MO, USA) and 2 mM L-glutamine (PanEco, Moscow, Russia). MSCs were maintained at 37 °C under 5% CO_2_, with the culture medium replaced every three days. Passage 3 murine MSCs were used to isolate EVs and CIMVs. The MSCs’ immunophenotype was determined using monoclonal antibodies to Sca-1–APC/Cy7 (108126; BioLegend, San Diego, CA, USA), CD49e–PE (1119525, Sony, New York, NY, USA), and CD44–APC/Cy7 (103028, BioLegend, USA). The expression of CD markers was analyzed by flow cytometry BD FACS Aria III (BD Bioscience, San Jose, CA, USA). The cells were found to express the following immunophenotype: Sca-1+, CD49e+, CD44+, which is characteristic of mouse MSCs, and CD45- as a negative control.

### 2.2. Preparation of EVs-Depleted Cell Culture Medium

FBS was ultracentrifuged at 100,000× *g* for 18 h at 4 °C using a BECKMAN L70 ultracentrifuge (Beckman Coulter, IN, USA). Then, the collected supernatant was used to prepare the cell culture medium. DMEM/F12 medium (PanEco, Moscow, Russia) was supplemented with 2 mM L-glutamine (PanEco, Moscow, Russia) and 10% EVs-depleted FBS (Sigma-Aldrich, St. Louis, MO, USA).

### 2.3. EVs Isolation

MSCs were grown to 80% monolayer density in an inCusaFeMCO-15AC incubator (SANYO Electric Co., Ltd., Osaka City, Osaka, Japan) at 37 °C in a humid atmosphere containing 5% CO_2_. Then, the MSCs culture was washed three times with PBS, EVs-depleted cell culture medium or serum-free medium was added, and the cells were cultivated for 48 h. Then, the cells were used for CIMVs production and conditioned medium was used for EVs isolation. Conditioned medium was collected and subsequently centrifuged at 300× *g* for 5 min, at 300× *g* for 10 min, at 2300× *g* for 25 min, and at 10,000× *g* for 45 min at 4 °C. The supernatant was then transferred to ultracentrifuge tubes and centrifuged at 100,000× *g* for 90 min at 4 °C using an SW28Ti rotor (Beckman Coulter, Indianapolis, IN, USA) in a BECKMAN L70 ultracentrifuge (Beckman Coulter, Indianapolis, IN, USA). The precipitates obtained after centrifugation at 2300× *g*, 10,000× *g*, and 100,000× *g* were analyzed.

### 2.4. CIMVs Production

CIMVs were prepared as described previously with modifications [28]. Cells collected from the previous step were washed twice with PBS and maintained in DMEM/F12 with 10 µg/mL cytochalasin B (C6762, Sigma-Aldrich, St. Louis, MO, USA) for 30 min (37 °C, 5% CO_2_). The concentration of cytochalasin B was chosen and the CIMVs production protocol was designed based on research data [24,29,30,31,32] and viability tests of cells [25]. Then, the cell suspension was vortexed vigorously for 30 s and subjected to sequential centrifugation at 300× *g* for 5 min, 300× *g* for 10 min, 2300× *g* for 25 min, 10,000× *g* for 45 min at 4 °C, and 100,000× *g* for 90 min at 4 °C. The precipitates obtained after centrifugation at 2300× *g*, 10,000× *g*, and 100,000× *g* were analyzed.

### 2.5. Flow Cytometry Analysis

The pellets obtained after EVs and CIMVs isolation were analyzed using flow cytometry. A mixture of calibration particles of 0.22, 0.45, 0.88, 1.34, and 3.4 μm (Cat. No. PPS-6K, Spherotech, Lake Forest, IL, USA) was used for the calibration of the BD FACS Aria III (BD Bioscience, San Jose, CA, USA). Analysis of the yield of EVs and CIMVs after a series of centrifugations at 2300× *g* for 25 min, 10,000× *g* for 45 min, and 100,000× *g* for 90 min was performed using a BD FACS Aria III flow cytometer (BD Bioscience, San Jose, CA, USA). Each sample was recorded within 60 s.

### 2.6. Scanning Electron Microscopy (SEM)

The EVs and CIMVs were fixed (10% formalin for 15 min), dehydrated using a graded alcohol series, and dried at RT. Prior to imaging, the samples were coated with gold/palladium in a Quorum T150ES sputter coater (Quorum Technologies Ltd., Lewes, UK). The slides were analyzed using a Merlin field emission scanning electron microscope (Carl Zeiss, Oberkochen, Germany).

### 2.7. Protein Concentration Measurement

The pellets obtained after EVs and CIMVs isolation were incubated in lysing solution (50 mM Tris-HCl pH 7.4, 1% NP 40, 0.5% sodium deoxychalate, 0.1% SDS, 150 mM NaCl, 2 mM EDTA, and 1 mM PMSF) for 30 min on ice. Then, the resulting mixture was centrifuged at 14,000 rpm for 15 min at 4 °C. The supernatant was used to determine the total protein concentration using the Pierce™ BCA Protein Assay Kit (ThermoScientific, Waltham, MA, USA) according to the manufacturer’s instructions.

### 2.8. Dynamic Light Scattering Analysis (DLS)

EVs and CIMVs were resuspended in 1 mL of PBS, which had been previously filtered through a filter with a pore diameter of 0.1 μm. The suspension was placed into disposable cuvettes, which were transferred to a ZetasizerNano ZS instrument (Malvern Instruments, Malvern, UK). The hydrodynamic diameter of EVs and CIMVs was measured in triplicate, and the negative control was PBS used for the resuspension of vesicles.

### 2.9. Nanoparticle Tracking Analysis (NTA)

NTA analysis was performed using a NanoSight LM-10 instrument (Malvern Instruments, UK). CMOS cameras C11440-50B with an FL-280 Hamamatsu Photonics (Shizuoka, Japan) image capture sensor were used as a detector. Measurements were taken in a special cuvette for aqueous solutions, equipped with a 405 nm laser (version CD, S/N 2990491) and a sealing ring made of Kalrez material. The temperature was taken with an OMEGA HH804 contact thermometer (Engineering, Inc., Stamford, CT, USA) for all measurements. Samples for analysis were detected and injected into the measuring cell with a 1 mL glass 2-piece syringe (tuberculin) through the Luer (Hamilton Company, Reno, NV, USA). To increase the statistical dose, the sample was pumped through the measuring chamber using a piezoelectric dispenser. Each sample was detected sequentially 6 times; the recording time was sequential and amounted to 60 s. For processing the footage of the Nanosight instrument, NTA 2.3 software applications (build 0033) were used as described previously [33,34]. The detailed work is currently presented in the Principle of Operation by B. Carr and A. Malloy [35]. The hydrodynamic size meter (Dh) was calculated with the measurement of the two-dimensional Einstein–Stokes equation [36].

### 2.10. Statistical Analysis

Statistical analysis was performed using Student’s 𝑡-test (GraphPad Software, San Diego, CA, USA) with a significance level of 𝑝 < 0.05.

## 3. Results

### 3.1. Characterization of EVs and CIMV

**Figure 1:**
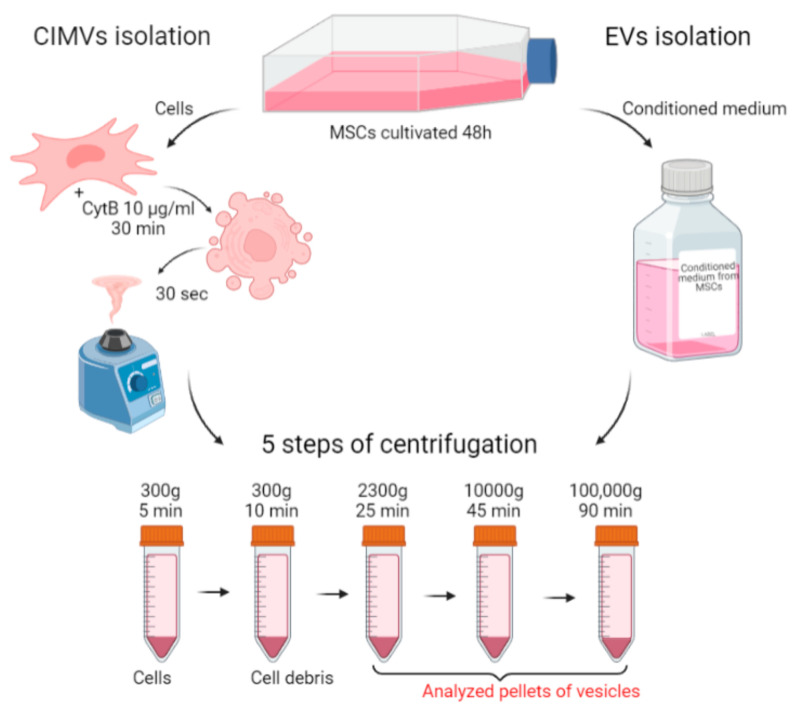
Scheme of the experiments. EVs and CIMVs were isolated from the same MSCs culture. Created with BioRender.com.

To standardize the conditions of the comparative analysis, the same culture was taken for the isolation of EVs and CIMVs. A scheme describing the isolation of EVs and CIMVs is presented in Figure 1. MSCs were cultivated in EVs-depleted medium for 48 h. Then, the conditioned medium was collected and used for EVs isolation, and the cells were collected and used for CIMVs production (Figure 1). Thus, we standardized the yield of EVs and CIMVs from an identical cell culture and the number of cells producing vesicles. The standard protocol of EVs isolation included the sedimentation of debris at low speeds of ~2000–20,000× *g* and ultracentrifugation at ~100,000–120,000× *g* [37,38]. Meanwhile, the maximum reported centrifugation rate for CIMVs isolation is 2000–2300× *g* [27]. We applied the following centrifugation mode for EVs and CIMVs isolation: 300× *g* for 5 min, 300× *g* for 10 min, 2300× *g* for 25 min, 10,000× *g* for 45 min at 4 °C, and 100,000× *g* for 90 min at 4 °C, which allowed further comparative analysis. The pellets obtained after centrifugation at 2300× *g* (further in the text—EVs 2300× *g* or CIMVs 2300× *g*), at 10,000× *g* (further in the text—EVs 10,000× *g* or CIMVs 10,000× *g*), and at 100,000× *g* (further in the text–EVs 100,000× *g* or CIMVs 100,000× *g*) were analyzed using the SEM, flow cytometry, protein analysis, DLS, and NTA methods.

**Figure 2:**
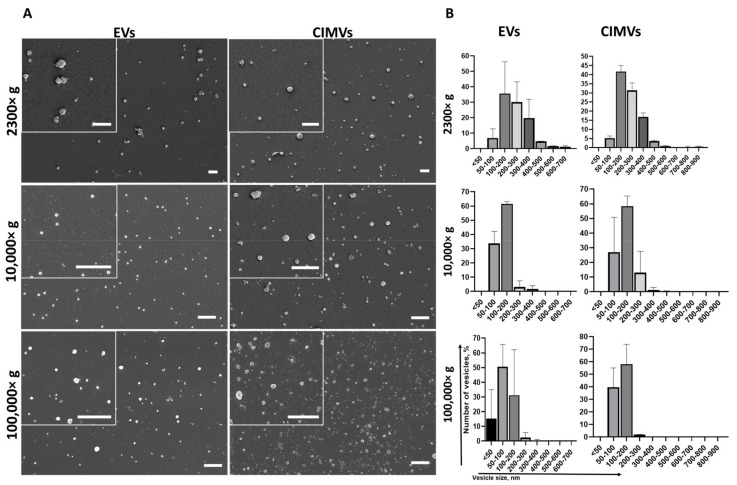
Analysis of morphology (**A**) and size distribution (**B**) of vesicles obtained during EVs and CIMVs isolation after centrifugation 2300× *g*, 10,000× *g*, and 100,000× *g*. Scale bar—1 µm.

We characterized the morphology and size of EVs and CIMVs by SEM. We found that EVs and CIMVs were rounded microstructures (Figure 2A). After centrifugation 2300× *g*, the EVs fraction contained particles from 50 nm to 700 nm in size, while CIMVs obtained at 2300× *g* were from 50 nm to 900 nm in size (Figure 2B). Centrifugation 10,000× *g* led to the sedimentation of EVs, with their size ranging from 50 nm to 400 nm, and CIMVs sedimentation, with their size ranging from <50 nm to 600 nm (Figure 2B). Applying ultracentrifugation 100,000× *g*, we pelleted EVs from <50 nm to 400 nm and CIMVs from <50 nm to 500 nm (Figure 2B).

**Figure 3:**
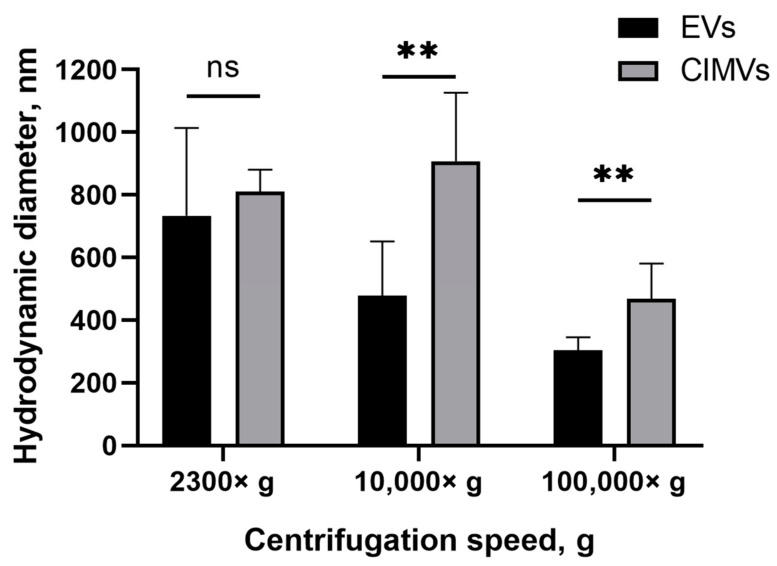
Average diameter of mouse EVs and CIMVs centrifuged at 2300× *g*, 10,000× *g*, and 100,000× *g*. Method—dynamic light scattering. The data represent mean ± SD. ns—not statistically significant. (**)—level of significance *p* < 0.01.

Next, we evaluated the size of the EVs and CIMVs by DLS. According to the results obtained by DLS, the average diameter of the EVs after centrifugation 2300× *g* was 733 ± 279 nm, whereas the average diameter of CIMVs was 810 ± 69 nm (Figure 3). The average diameter of EVs after centrifugation 10,000× *g* was 478 ± 173 nm, CIMVs—907 ± 219 nm (Figure 3). Centrifugation at 100,000× *g* led to the sedimentation of EVs with an average diameter of 303 ± 41 nm, and CIMVs with an average diameter of 467 ± 112 nm (Figure 3). The original histograms obtained after DLS analysis of EVs and CIMVs are provided in the Supplementary Materials (Figure S1). According to the obtained DLS histograms, CIMVs were more homogeneous in size compared with EVs.

The NTA method showed that the average diameters of EVs and CIMV were similar at all centrifugation steps: EVs sedimented at 2300× *g* were 118 ± 62 nm in size and CIMVs were 120 ± 67 nm, the EVs sedimented at 10,000× *g* were 116 ± 67 nm and CIMVs were 118 ± 66 nm, and EVs sedimented at 100,000× *g* were 92 ± 60 nm and CIMVs were 97 ± 56 nm.

### 3.2. Yield of EVs and CIMVs

**Figure 4:**
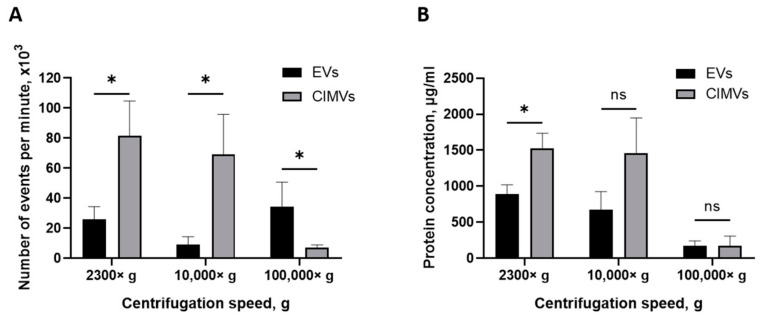
Analysis of EVs and CIMVs yield. (**A**) Average number of EVs and CIMV after centrifugation 2300× *g*, 10,000× *g*, and 100,000× *g*. Flow cytometry method. (**B**) Total protein concentrations of EVs and CIMVs after centrifugation 2300× *g*, 10,000× *g*, and 100,000× *g*. Bicinchoninic acid method. The data represent mean ± SD. ns—not statistically significant. (*)—level of significance *p* < 0.05.

The yield of EVs and CIMVs was evaluated by quantifying the amount of vesicles using flow cytometry and by determining the total protein concentration. Pellets obtained after centrifugation 2300× *g*, 10,000× *g*, and 100,000× *g* were resuspended in an equal volume of PBS and analyzed by a flow cytometer with an enhanced detector. We found that the average numbers of EVs and CIMVs obtained after centrifugation 2300× *g* were 26,000 ± 8485 events/min and 81,666 ± 23,116 events/min, respectively (*p* = 0.015), 10,000× *g* were 9333 ± 5132 events/min and 69,000 ± 26,870 events/min, respectively (*p* = 0.026), and 100,000× *g* were 34,500 ± 16,263 events/min and 7300 ± 1697 events/min, respectively (*p* = 0.27) (Figure 4A).

One of the most popular methods for expressing the amount of EVs is the total protein concentration [39]. Therefore, we also evaluated the yield of vesicles by determining the total protein concentration. We found that the average yields of EVs and CIMVs obtained after centrifugation at 2300× *g* were 888.12 ± 131 µg/mL and 1525.59 ± 210 μg/mL in total protein concentration, respectively (*p* = 0.02), at 10,000× *g* were 673.27 ± 254 µg/mL and 1455.94 ± 493 μg/mL, respectively (*p* = 0.09), and at 100,000× *g* were 175.10 ± 65 μg/mL and 175.13 ± 132 μg/mL, respectively (*p* = 0.99) (Figure 4B). The obtained results are shown in Table 1. 

### 3.3. Amount of EVs and CIMVs Analyzed by NTA

**Figure 5:**
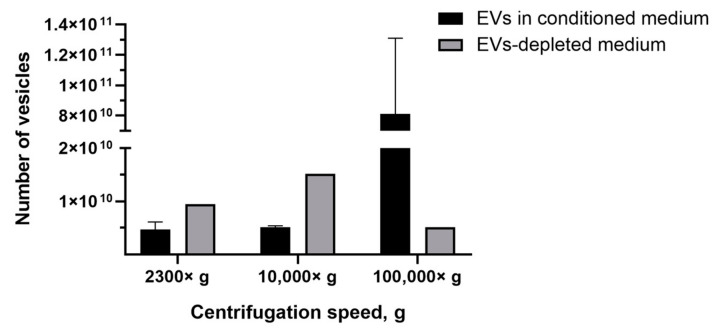
Analysis of the number of EVs isolated from conditioned medium and the number of EVs-like particles isolated from the EVs-depleted medium. NTA method.

The most common tool for EVs quantification in solution is NTA. Recently, it has been shown by NTA that EVs-like particles remain in FBS, despite overnight ultracentrifugation [40]. Therefore, we determined the number of EVs-like particles from the EVs-depleted medium, which affected the analysis by introducing an overestimation error. We found that, after centrifugation 2300× *g*, the number of pelleted EVs-like particles was 9.5 × 10^9^ particles/mL, whereas the average number of EVs isolated from conditioned medium (based on the EVs-depleted medium) was 4.7 ± 1.4 ×10^9^ particles/mL (Figure 5). After the centrifugation of the EVs-depleted medium 10,000× *g*, 1.5 × 10^10^ particles/mL of EVs-like particles were detected, and only 5.1 ± 0.3 × 10^9^ particles/mL of EVs were precipitated from the conditioned medium (based on EVs-depleted medium) (Figure 5). The number of EVs isolated from the conditioned medium exceeded the number of EVs-like particles from the EVs-depleted medium only when centrifuged at 100,000× *g*—8.1 ± 5 × 10^10^ particles/mL and 5.1 × 10^9^ particles/mL, respectively (Figure 5).

**Figure 6:**
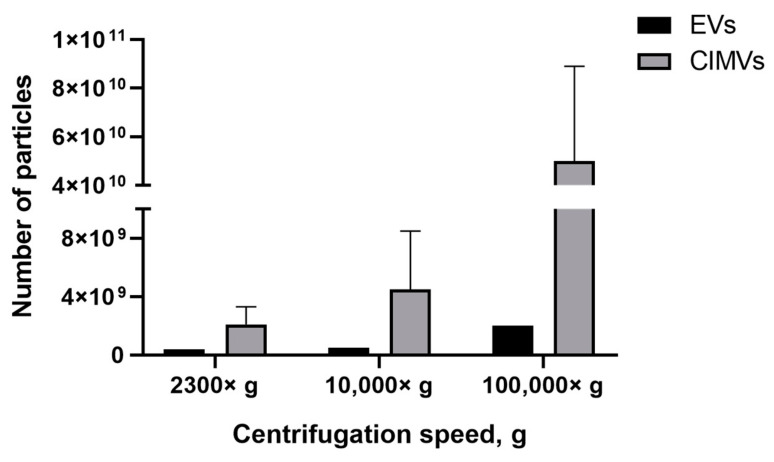
Analysis of EVs and CIMVs yields. Average number of EVs and CIMV after centrifugation 2300× *g*, 10,000× *g*, and 100,000× *g*. NTA method.

We found that the EVs-depleted medium contained a significant number of EVs-like particles, which can affect the yield analysis results. Therefore, we used serum-free medium to culture MSCs and collect condition medium for the subsequent EVs isolation. According to the experimental scheme (Figure 1), conditioned medium (based on serum-free medium) was collected from the MSCs culture and cells were used in the CIMVs production protocol. We observed that the number of EVs isolated at 2300× *g* was 0.4 × 10^9^ particles/mL, whereas the CIMVs number was 2.1 ± 1.2 × 10^9^ particles/mL; 10,000× *g*, the number of EVs was 0.5 × 10^9^ particles/mL and that of CIMVs was 4.5 ± 4 × 10^9^ particles/mL (Figure 6). After centrifugation 100,000× *g*, the difference in the yield became more tremendous—2 × 10^9^ particles/mL of EVs and 5 ± 3.9 × 10^10^ particles/mL of CIMVs (Figure 6).

## 4. Discussion

Extracellular vesicles have become popular over the past 10 years. Their potential has long been underestimated due to the lack of knowledge and methods for detecting nanosized particles [41]. To date, with the improvement in the quality of methods for their detection and isolation, interest in EVs has increased significantly. One of the obstacles to the translation of EVs to the clinic is their low yield. The treatment of cells with cytochalasin B has been shown to be effective for the generation of vesicles in terms of retaining cell plasma membrane receptors, the cytoplasmic content, and biological activity of parental cells [24,27].

We previously showed that treatment with cytochalasin B leads to the disorganization of the cytoskeleton and rounding of cells [25]. Here, we performed an additional experiment to evaluate the effect of cytochalasin B on the phenotype and viability of MSCs. We found that, after 30 min of incubation with cytochalasin B, the cells became rounded with some cytoplasmic outgrowths attached to the culture dish left (Figure S2, Supplementary Material), and no impairment of cell viability was observed (Figure S3, Supplementary Material). Next, we evaluated the effect of cytochalasin B treatment and vortexing separately on the phenotype and viability of cells (Figures S3 and S4, Supplementary Material). We found that the vortexing of native cells resulted in the appearance of debris (Figure S4B, Supplementary Material), while pretreatment with cytochalasin B followed by vortexing caused the pinching off of small, rounded structures (membrane vesicles) (Figure S4D, Supplementary Material). Moreover, the vortexing of native cells (without pretreatment with cytochalasin B) led to an increase in the percent of apoptotic cells (statistically non-significant) (Figure S3E, Supplementary Material), and pretreatment with cytochalasin B followed by vortexing did not affect the viability of MSCs (Figure S3, Supplementary Material). We believe that the vortexing of native MSCs leads to cell damage due to the presence of a rigid cytoskeleton. Meanwhile, pretreatment of cells with cytochalasin B makes them more plastic and allows membrane vesicles to pinch off under vortexing. Therefore, the pretreatment of cells with cytochalasin B is a necessary condition for membrane vesicles production.

Next, for the first time, we compared the size, morphology, and yield of EVs and CIMS derived from murine MSCs. In order to maintain accuracy in the comparative analysis, the same culture was used for the isolation of EVs and CIMVs: conditioned medium was used for EVs isolation and the cells were harvested for CIMVs production.

To compare EVs and CIMVs, we applied the most commonly employed protocols for EVs isolation and included one intermediate step with the centrifugation rate for CIMVs isolation: 300× *g* for 5 min, 300× *g* for 10 min, 2300× *g* for 25 min, 10,000× *g* for 45 min at 4 °C, and 100,000× *g* for 90 min at 4 °C. The pellets obtained after centrifugation 2300× *g*, 10,000× *g*, and 100,000× *g* were analyzed.

We used SEM as the most accurate technique to characterize the morphology and determine the size distribution of EVs and CIMVs. We found that the majority of EVs and CIMVs obtained at 2300× *g* had similar sizes, from 100–400 nm. Differences in the size of EVs and CIMVs were observed after centrifugation at 10,000× *g*—the number of larger vesicles (200–300 nm in size) in the CIMVs population increased by 9.9% compared with EVs (Figure 2B). The observed difference may have been due to the fact that CIMVs are not formed naturally, but by chemical and mechanical action on the producer cells [23]. As a result, naturally formed and induced vesicles differ in size. After centrifugation at 100,000× *g*, the number of vesicles smaller than 50 nm in the EVs population was increased by 15% compared with CIMVs (Figure 2B). The observed difference may have been due to the fact that the EVs population includes exosomes (EVs with size from less than 50–100 nm), which are pelleted at 100,000× *g*. Meanwhile, CIMVs are formed by pinching off from the cell’s surface.

SEM is a highly labor-intensive technique. Besides electron microscopy, flow cytometry, protein quantification, DLS, and NTA are widely used for EVs quantification. According to the DLS results, EVs and CIMVs isolated at 2300× *g* did not differ in their average hydrodynamic diameter (Figure 3). The EVs obtained after centrifugation at 10,000× *g* were 1.9 times smaller in size than CIMVs (*p* = 0.004), and 1.54 times smaller in size than the CIMVs obtained after centrifugation at 100,000× *g* (*p* = 0.009) (Figure 3). We believe that the median diameter of CIMVs being greater than that of EVs is due to the fact that: (1) CIMVs are mostly cytoplasmic membrane-derived vesicles (which are large in size), whereas EVs include small vesicles of endosomal origin—exosomes (30–100 nm in size); and (2) CIMVs production is induced by chemical and physical methods in contrast to the natural process of EVs release.

In contrast to the SEM data, the average diameter of EVs and CIMVs determined by DLS was greater (Figure 2B and Figure 3). This difference may have been due to the DLS limitation, connected to the biased detection of larger particles [36]. We found that the DLS and NTA vesicles size data were significantly different (for example, the average diameter of EVs after centrifugation at 100,000× *g* was 303 ± 41 nm according to DLS and 92 ± 60 nm according to NTA). We believe that this discrepancy was due to the high polydispersity of vesicles and methodological differences between the DLS and NTA methods. Compared with DLS, NTA has higher resolving capabilities for analyzing particles with a mean diameter of less than 100 nm [42]. To analyze polydisperse samples, such as EVs, the NTA system should be equipped with three variable light sources (450 nm, 520 nm, and 635 nm) [42]. Meanwhile, our system was equipped with a 405 nm laser only. As each of the used methods has its pros and cons connected to the biased detection of large or small particles, we compared the obtained data and concluded that EVs ranged in size from <50 nm to 1012 nm, and CIMVs ranged from <50 to 1126 nm.

Next, we investigated the yield of vesicles by flow cytometry. We found that the amount of CIMVs exceeded the amount of EVs after centrifugation 2300× *g* by 3.14 times (*p* = 0.05) and after centrifugation 10,000× *g* by 7.39 times (*p* = 0.027) (Figure 4A). However, after centrifugation 100,000× *g*, the amount of EVs tended to exceed the amount of CIMVs by 4.7 times (statistically non-significant) (Figure 4A). Thus, we found that the protocol of cells treatment with cytochalasin B increased the yield of vesicles (at 2300× *g* and 10,000× *g*) and allowed vesicles production in a short time. The tendency to detect more EVs after centrifugation 100,000× *g* might have been due to the presence of small vesicles (exosomes) in the EVs sediment. As we demonstrated above, the EVs-like particles from the EVs-depleted medium could have contributed to the overestimation of the number of EVs. At the same time, this circumstance does not affect the accuracy of calculating the number of CIMVs, as only washed cells are used for CIMVs production.

We analyzed the yield of EVs and CIMVs in the protein content. The amount of CIMVs in the total protein content exceeded the amount of EVs obtained after centrifugation 2300× *g* by 1.71 times (*p* = 0.023) and that obtained after centrifugation 10,000× *g* by 2.16 times (statistically non-significant). The protein concentration data are consistent with the data obtained by flow cytometry, where an increased CIMVs yield compared with EVs was observed after centrifugation 2300× *g* and 10,000× *g*. No difference in the total protein concentration of CIMVs and EVs obtained after centrifugation 100,000× *g* was observed (Figure 4B). In contrast, flow cytometry data indicated that the yield of EVs tended to exceed the amount of CIMVs. This observed inconsistency may have been due to the fact that, after centrifugation 100,000× *g*, the EVs with a smaller size were sedimented (SEM and DLS data) (Figure 2 B and Figure 3). Therefore, despite the higher number of EVs sedimented after centrifugation 100,000× *g*, their amount in the total protein concentration did not exceed the CIMVs concentration.

Next, we compared the obtained results with data obtained by the NTA method which is the most common tool for EVs quantification. NTA allows visualization in real-time and the calculation of the size and concentration of particles based on light scattering and the Brownian movement [43]. In the first stage, we determined the impact of contamination with lipoproteins and other aggregates of EV-like sizes on EVs quantification. After centrifugation 2300× *g* and 10,000× *g*, a comparable or higher number of EV-like particles over EVs was detected (Figure 5). After centrifugation at 100,000× *g*, the number of EV-like particles was 5.1 × 10^9^ particles/mL (Figure 5). Despite the number of EVs exceeding the number of EV-like particles ten times, we found that the presence of residual EVs-like particles in the EVs-depleted medium introduced a significant inaccuracy in the calculation of the EVs yield. Therefore, we cultivated cells in a serum-free medium for subsequent EVs isolation. We observed that the number of CIMVs significantly exceeded the number of EVs after each step of centrifugation (2300× *g*, 10,000× *g*, and 100,000× *g*) by 5, 9, and 20 times, respectively (Figure 6).

## 5. Conclusions

The treatment of cells with cytochalasin B combined with mechanical action leads to membrane vesicles production. We applied the most commonly used methods of EVs evaluation—the SEM, flow cytometry, protein analysis, DLS, and NTA methods—to conduct a comprehensive comparative analysis. We demonstrated that, in contrast to the SEM data, the average diameter of EVs and CIMVs determined by DLS was larger due to the method’s limitations. We found that flow cytometry data and protein quantification data could be contradictory at first sight. However, the contradiction could be resolved if an additional analysis method is used, such as SEM. We found that EVs-like particles remained in FBS despite overnight ultracentrifugation, which introduced a significant inaccuracy in the calculation of the EVs yield. We observed that the number of CIMVs significantly exceeded the number of EVs after each step of centrifugation (2300× *g*, 10,000× *g*, and 100,000× *g*) by up to 5, 9, and 20 times, respectively.

## Figures and Tables

**Table 1:** Yield and protein concentration of EVs and CIMVs.

	EVs		CIMVs	
	Yield, Events/min	Protein, µg/mL	Yield, Events/min	Protein, µg/mL
2300× *g*	26,000 ± 8 485	888.12 ± 131	81,666 ± 23 116	1525.59 ± 210
10,000× *g*	9333 ± 5 132	673.27 ± 254	69,000 ± 26 870	1455.94 ± 493
100,000× *g*	34,500 ± 16 263	175.10 ± 65	7300 ± 1 697	175.13 ± 132

## Data Availability

All data generated or analyzed during this study are included in this published article. The data that support the findings of this study are available from the corresponding author upon request.

## Supplementary Materials

The following supporting information can be downloaded at: https://www.mdpi.com/article/10.3390/cimb45030158/s1, Figure S1: Dynamic light scattering profiles of EVs and CIMVs; Figure S2: Analysis of phenotype of native mouse MSCs or treated with cytochalasin B for 30 min; Figure S3: Dot plots depicting native mouse MSCs and after 30 min incubation with cytochalasin B analyzed by flow cytometry; Figure S4: Analysis of mouse MSCs phenotype in suspension: native MSCs and after 30 minutes incubation with cytochalasin B.
