# Supplementary material for: Increased Yield of Extracellular Vesicles after Cytochalasin B Treatment and Vortexing
Source: Curr Issues Mol Biol. 2023 Mar 15;45(3):2431–43. doi: 10.3390/cimb45030158 (PMC10047179; doi:10.3390/cimb45030158)
Supplement: Supplementary file 1 [file cimb-45-00158-s001.zip › cimb-2084668-supplementary.pdf]

Supplementary materials:

Increased Yield of Extracellular Vesicles after Cytochalasin B Treatment and Vortexing

Sirina V. Kurbangaleeva, Valeriia Y. Syromiatnikova, Angelina E. Prokopeva, Aleksey M. Rogov, Artur A. Khannanov, Albert A. Rizvanov and Marina O. Gomzikova *

1. DLS analysis

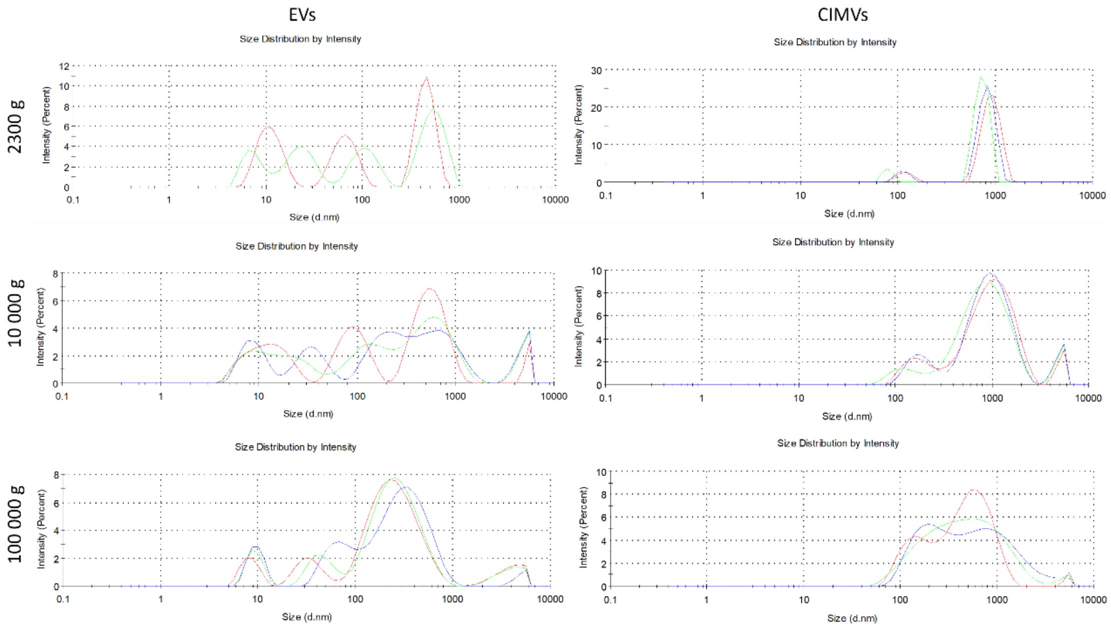

Figure S1. Dynamic light scattering profiles of EVs and CIMVs.

Citation: Kurbangaleeva, S.V.; Syromiatnikova, V.Y.; Prokopeva, A.E.; Rogov, A.M.; Khannanov, A.A.; Rizvanov, A.A.; Gomzikova, M.O. Increased Yield of Extracellular Vesicles after Cytochalasin B Treatment and Vortexing. *Curr. Issues Mol. Biol.* **2023**, *3*, x. https://doi.org/10.3390/xxxxx

Academic Editors: Gerhard Breves

Received: 23 November 2022

Revised: 7 March 2023

Accepted: 13 March 2023

Published: 15 March 2023

Copyright: © 2023 by the authors. Submitted for possible open access publication under the terms and conditions of the Creative Commons Attribution (CC BY) license (https://creativecommons.org/licenses/by/4.0/).

2. Impact of cytochalasin B treatment on phenotype of adherent MSCs

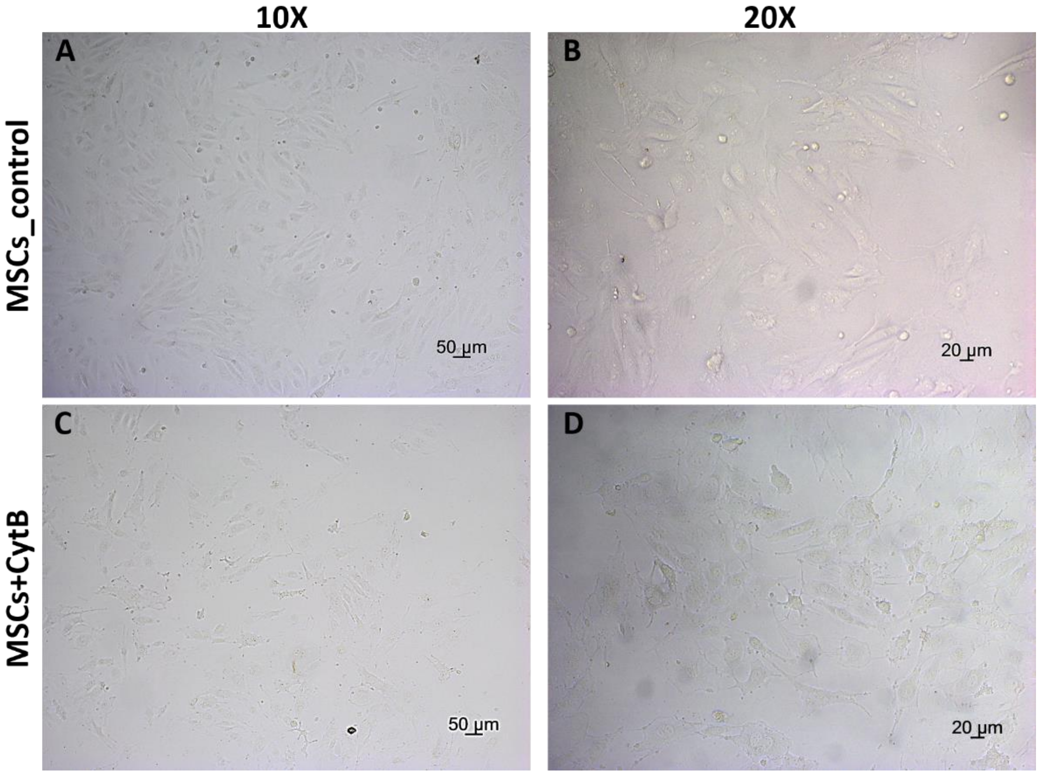

Figure S2. Analysis of phenotype of native mouse MSCs (A,B) or treated with cytochalasin B for 30 min (C,D). Light microscopy AxioOberver.Z1 (Carl Zeiss, USA). Magnification 10X (A,C) and 20X (B,D).

3. Impact of cytochalasin B treatment and vortexing on MSCs viability.

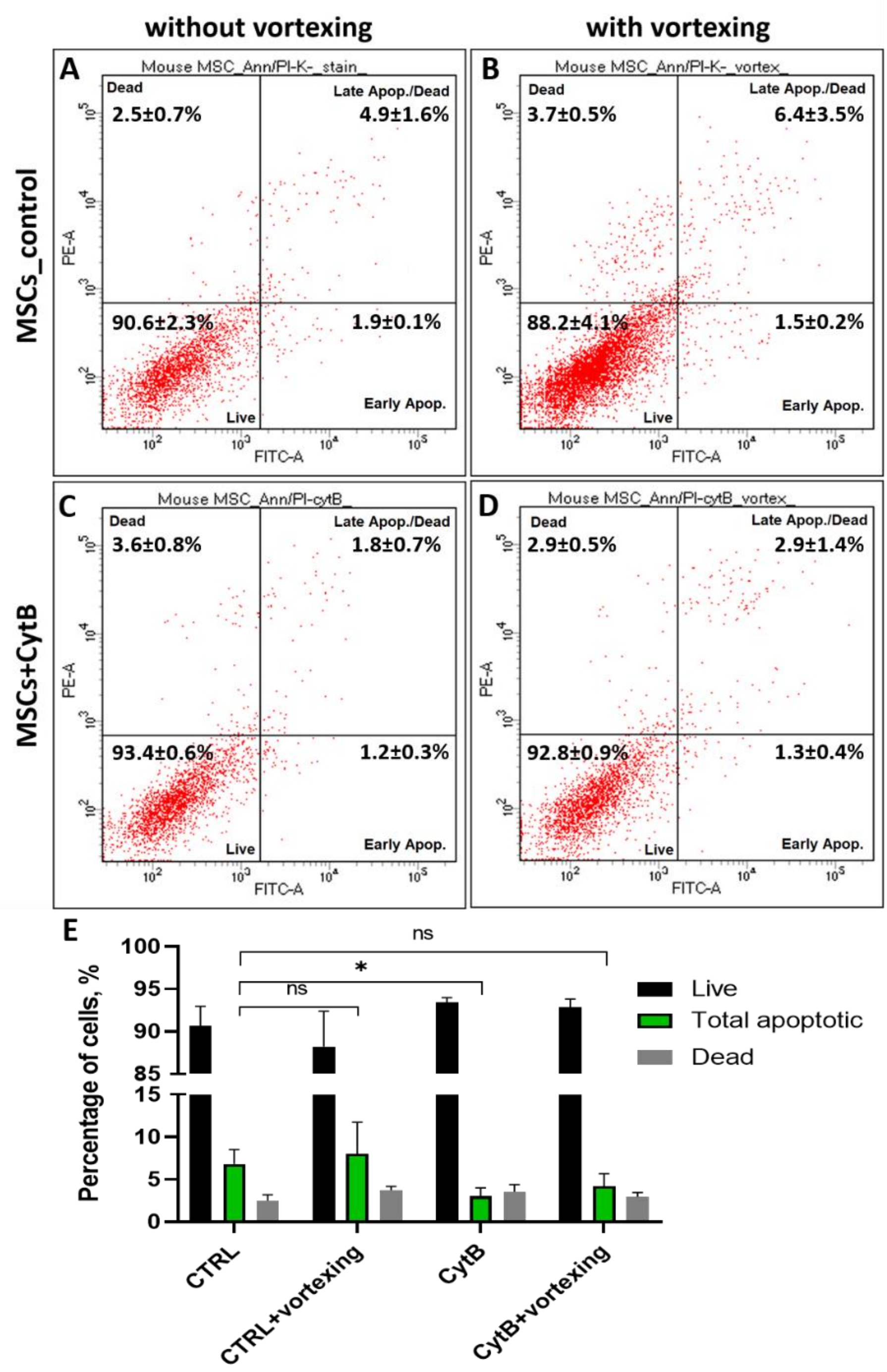

Figure S3. Dot plots depicting native mouse MSCs (A,B) and after 30 min incubation with cytochalasin B (C,D) analyzed by flow cytometry BD FACS Aria III (BD Bioscience, USA). Analysis of percentage of live (black), apoptotic (green) and dead (gray) MSCs (E). ns—not statistically significant. (*)—level of significance $p < 0.05$.

4. Effect of cytochalasin B treatment and vortexing on MSCs phenotype in suspension

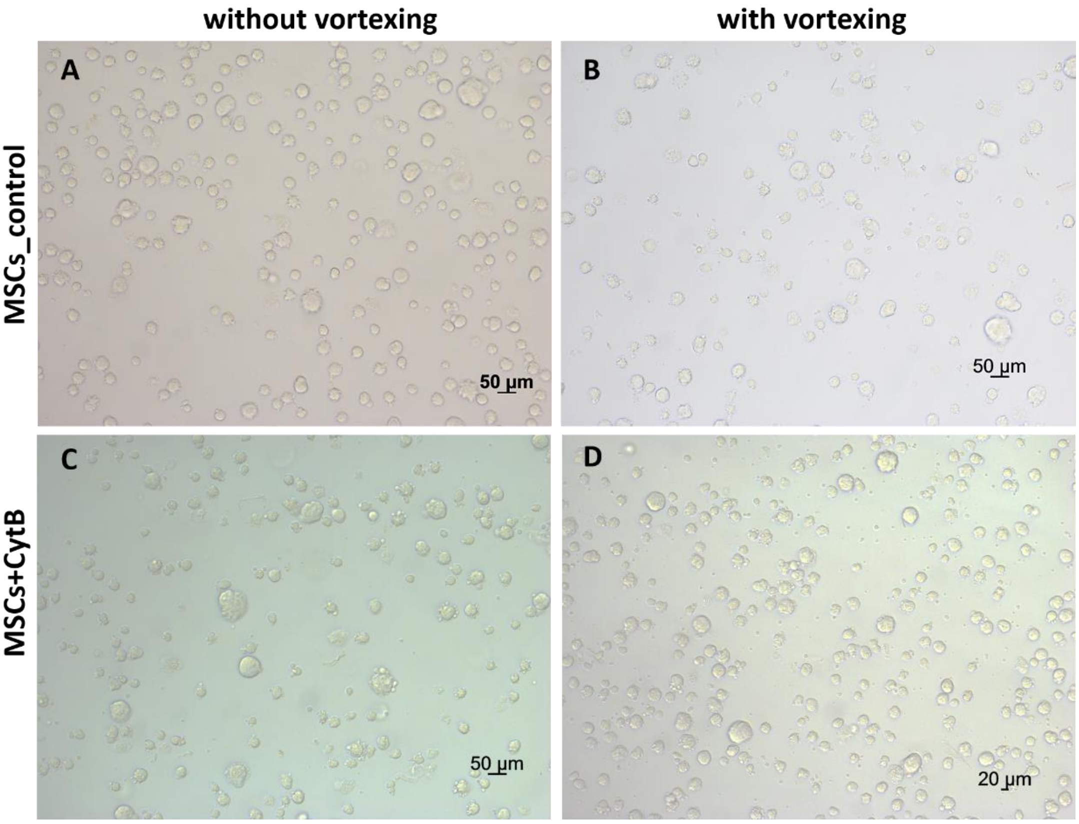

Figure S4. Analysis of mouse MSCs phenotype in suspension: native MSCs (A,B) and after 30 min incubation with cytochalasin B (C,D). Light microscopy AxioOberver.Z1 (Carl Zeiss, USA). Magnification 20X.
